# Beyond temperature: Why climate adaptation in agriculture needs a systems approach

**DOI:** 10.1073/pnas.2614201123

**Published:** 2026-06-01

**Authors:** Bruno Basso

**Affiliations:** ^a^ Department of Earth and Environmental Sciences, Michigan State University, East Lansing, MI 48824; ^b^ W.K. Kellogg Biological Station, Michigan State University, Hickory Corners, MI 49060; ^c^ Great Lakes Bioenergy Research Center, Michigan State University, East Lansing, MI 48824

Few questions cast a longer shadow over agricultural sustainability than the question of how rising temperatures will reshape the balance among crops, pests, and the beneficial insects that suppress them. For more than a decade, the dominant projection has been based on the assumption that warmer climates will drive the proliferation of crop pests worldwide, decimate natural enemies, and simultaneously increase pesticide use and decrease yield. In PNAS, Lippey et al. (1) confront that projection with an unusually powerful body of evidence—141,562 field‐year observations of 43 arthropod populations across 13,308 sites and 13 y in two temperate agricultural regions. The message is clear and, for the field, important. Some populations increase with warming, others decline, many show no detectable response, and laboratory‐measured thermal performance traits fail to predict which species fall into which camp.

This is an exemplary piece of system ecology. The authors leverage three long‐term monitoring databases from Andalusia and California to do what a single experiment never could. They tested the pest‐proliferation hypothesis at the scale, duration, and environmental realism for which it is intended. They validate their pest‐density results against pesticide application intensity, an independent, economically grounded signal of growers’ actual perception of pest pressure. They separate pests from natural enemies and show that winter warming poses the deeper threat to biological control services, a result with immediate implications for Mediterranean and temperate agroecosystems. And they close with an honest reckoning: laboratory‐derived thermal optima and life‐history traits, read one at a time, do not predict field outcomes.

“In PNAS, Lippey et al. (1) confront that projection with an unusually powerful body of evidence—141,562 field‐year observations of 43 arthropod populations across 13,308 sites and 13 y in two temperate agricultural regions.”

The significance of this work is not in rejecting climate‐driven pest risk. Risk remains and will grow. The paper’s significance is that it dismantles the convenient story of a single‐driver, species‐agnostic response and in doing so, exposes a more uncomfortable truth about how we forecast the biosphere under climate change.

What Lippey et al. call the failure of thermal mismatch to predict field responses is, in the broader agroecological context, entirely expected. Temperature in the field is never delivered alone. It arrives coupled to rainfall patterns, soil moisture dynamics, vapor‐pressure deficit, radiation, elevated atmospheric CO_2_, and the cascading changes these drive in host‐plant chemistry, phenology, and architecture. Any of these can be the rate‐limiting variable in any given year, field, or life stage, and their relative importance shifts across the growing season.

Consider rainfall alone. Across the US Corn Belt, year‐to‐year variation in yield stability is driven more by water availability and its interaction with soil texture than by temperature per se (2). In the Mediterranean systems where Lippey et al. worked, extended drought alters host‐plant nitrogen status, trichome density, and secondary chemistry—all of which modify herbivore performance independently of insect body temperature. Soil water limitation changes microclimate at the leaf and canopy surfaces where most herbivores actually live (3); elevated CO_2_ dilutes foliar nitrogen, slowing some chewing insects while accelerating some phloem feeders (4). Add to this a warming winter that advances green‐up by 2 wk, a landscape where pesticide practices have shifted in response to resistance, a predator community restructured by a dry summer, and the laboratory‐predicted response curve loses its prognostic value. It becomes a component of a system, not the system itself.

Lippey et al.’s own conceptual figure 2 captures this beautifully. The striking asymmetry between their panels A (laboratory—one axis) and B (field—a constellation of interacting drivers) is, in my view, the most important image in the paper. It applies far beyond pest ecology. It is a diagnosis of why almost every single‐driver forecast of agroecosystem behavior under climate change has struggled to match field observations, and it generalizes (Fig. 1 of this commentary) across every level of agricultural sustainability assessment.

**Fig. 1.**
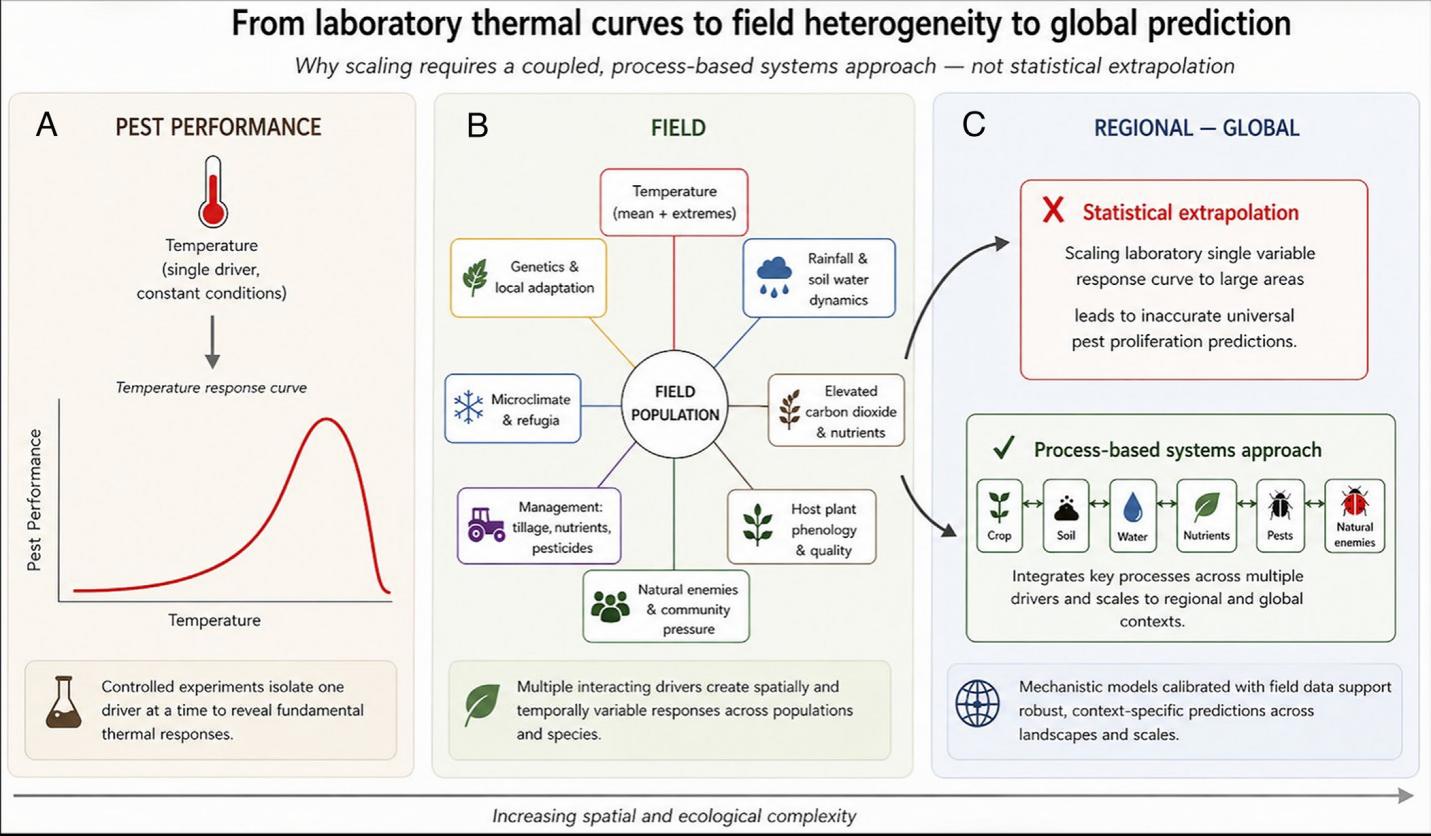
From laboratory thermal curves to field heterogeneity to global prediction. (*A*) Laboratory thermal performance experiments isolate temperature as the sole driver of pest performance, yielding tidy dome‐shaped curves under constant conditions. (*B*) In the field, populations are embedded in a web of interacting drivers—rainfall and soil water, elevated CO_2_ and tissue nitrogen, host‐plant phenology, natural‐enemy pressure, community pressure, management, microclimate, local adaptation—producing heterogeneous, largely linear responses as documented by Lippey et al. (*C*) Scaling to regional and global predictions cannot proceed by statistical extrapolation of single‐driver curves. It requires coupled, process‐based models that represent the full biophysical system, are calibrated on field data, and are validated regionally.

The authors’ natural‐enemy finding deserves particular attention. Contrary to the prevailing expectation, summer responses did not differ significantly between pests and their enemies. But winter minimum temperatures and annual means did. The natural enemies declined where pests increased. Because winter temperatures are warming roughly twice as fast as summer temperatures (5), this is not a peripheral observation. It identifies the season and mechanism through which climate change is most likely to erode biological control, precisely when monitoring networks are least active and farmer attention is lowest.

This seasonal asymmetry also carries a water‐budget dimension that the paper touches on only briefly. Warmer winters in Mediterranean systems shorten the recharge window during which soils refill their water storage. Reduced soil water at spring green‐up compresses the phenological window during which omnivorous predators can supplement their diet with plant tissues before arthropod prey become available. What looks like a temperature effect on overwintering natural enemies is often, mechanistically, a coupled temperature–precipitation–soil water effect transmitted through the plant community. Untangling it demands models that resolve soil water, plant growth, and arthropod phenology at a daily time step, which is to say, process‐based cropping‐system models of the kind that have become the backbone of climate‐adaptation science for crops themselves (6).

## Scaling Requires a Systems Approach, Not a Statistical Extrapolation

Herein lies the deeper methodological implication of Lippey et al.’s results. The trait‐based frameworks they test follow a specific logic: Measure a species’ thermal response in the laboratory, compute its mismatch with projected ambient temperature, and extrapolate the difference into a forecast. That logic assumes temperature is separable from the rest of the system. The field data say it is not. And the failure of that logic has direct parallels in other domains of agriculture, for instance soil organic carbon dynamics cannot be predicted from a single‐site incubation curve (7, 8); N_2_O emissions cannot be estimated from a fixed percentage of applied nitrogen (9); regional yield stability cannot be projected from yield‐temperature regressions that ignore hydrology, management, and soil heterogeneity (2, 10). In every case, the single‐driver extrapolation looks deceptively tractable at the scale of a publication and deceptively wrong at the scale of a landscape.

The alternative is not more traits or larger laboratory experiments. It is a systems approach that is able to capture the species specific dynamics under different pedoclimatic conditions. Concretely, this means process‐based models that represent the full biophysical system (soil, water, crop, climate, and the biotic community). The models need to be validated against field‐measured outputs rather than laboratory‐measured parameters alone and should be run against dynamic counterfactual baselines so that the attribution of change is accurate, and if feasible deployed in multimodel ensembles where structural uncertainty is large. This is the architecture that has moved crop climate‐adaptation science from statistical extrapolation to credible, region‐specific projection (6, 9). Extending it to coupled crop–pest–natural‐enemy dynamics is the obvious next step, and Lippey et al.’s dataset is precisely the kind of calibration resource such a program needs.

The 141,562 observations assembled here, the 13 y of pesticide decisions, the 43 population response coefficients across species, crops, and regions are critical validation data without which models are speculation and with them they become prediction. Laboratory experiments reveal mechanism while field monitoring reveals realized outcomes; process‐based models reconcile the two across space and time. Lippey et al.’s paper should be read as a call for that three‐way partnership, not as a rejection of the laboratory program that generated the original thermal performance curves.

We should aim to invest in more intensive species‐specific monitoring networks for pest and their natural enemies, of the kind that made this paper possible. Climate‐adaptation science for agriculture must move decisively away from single‐driver, trait‐based extrapolation and toward multidriver, process‐based, field‐validated system models. Pests, soil, water, nutrients, and yield are not separable problems. They are coupled states of the same system, and the only credible forecasts will come from frameworks that treat them that way.

Lippey et al. do not resolve the fate of agricultural arthropods under climate change; they reframe the question. They show that universal predictions were always going to fail because the real world refuses to be universal. They also show that the scientific infrastructure exists to replace those predictions with something better, large‐scale, long‐term field data, analyzed with proper statistics, and coupled with process‐based models that can project them forward.
